# Genome Size, Karyotype Polymorphism and Chromosomal Evolution in *Trypanosoma cruzi*

**DOI:** 10.1371/journal.pone.0023042

**Published:** 2011-08-12

**Authors:** Renata T. Souza, Fábio M. Lima, Roberto Moraes Barros, Danielle R. Cortez, Michele F. Santos, Esteban M. Cordero, Jeronimo Conceiçao Ruiz, Samuel Goldenberg, Marta M. G. Teixeira, José Franco da Silveira

**Affiliations:** 1 Departamento de Microbiologia, Imunologia e Parasitologia, Escola Paulista de Medicina, Universidade Federal de São Paulo, São Paulo, Brazil; 2 Centro de Pesquisas Rene Rachou, Fiocruz, Minas Gerais, Brazil; 3 Instituto Carlos Chagas, Paraná, Brazil; 4 Departamento de Parasitologia, Instituto de Ciências Biomédicas, Universidade de São Paulo, São Paulo, Brazil; Universidade de Sao Paulo, Brazil

## Abstract

**Background:**

The *Trypanosoma cruzi* genome was sequenced from a hybrid strain (CL Brener). However, high allelic variation and the repetitive nature of the genome have prevented the complete linear sequence of chromosomes being determined. Determining the full complement of chromosomes and establishing syntenic groups will be important in defining the structure of *T. cruzi* chromosomes. A large amount of information is now available for *T. cruzi* and *Trypanosoma brucei*, providing the opportunity to compare and describe the overall patterns of chromosomal evolution in these parasites.

**Methodology/Principal Findings:**

The genome sizes, repetitive DNA contents, and the numbers and sizes of chromosomes of nine strains of *T. cruzi* from four lineages (TcI, TcII, TcV and TcVI) were determined. The genome of the TcI group was statistically smaller than other lineages, with the exception of the TcI isolate Tc1161 (José-IMT). Satellite DNA content was correlated with genome size for all isolates, but this was not accompanied by simultaneous amplification of retrotransposons. Regardless of chromosomal polymorphism, large syntenic groups are conserved among *T. cruzi* lineages. Duplicated chromosome-sized regions were identified and could be retained as paralogous loci, increasing the dosage of several genes. By comparing *T. cruzi* and *T. brucei* chromosomes, homologous chromosomal regions in *T. brucei* were identified. Chromosomes Tb9 and Tb11 of *T. brucei* share regions of syntenic homology with three and six *T. cruzi* chromosomal bands, respectively.

**Conclusions:**

Despite genome size variation and karyotype polymorphism, *T. cruzi* lineages exhibit conservation of chromosome structure. Several syntenic groups are conserved among all isolates analyzed in this study. The syntenic regions are larger than expected if rearrangements occur randomly, suggesting that they are conserved owing to positive selection. Mapping of the syntenic regions on *T. cruzi* chromosomal bands provides evidence for the occurrence of fusion and split events involving *T. brucei* and *T. cruzi* chromosomes.

## Introduction

*Trypanosoma cruzi* is a protozoan parasite transmitted to vertebrate hosts by insect vectors causing Chagas disease, also known as American trypanosomiasis. The disease is endemic in Latin America and affects approximately eight million people [1], with an increasing number of cases in non-endemic countries including the United States and Europe [2], [3]. The disease has a broad spectrum of clinical symptoms, which may reflect parasite and host genetic factors. *T. cruzi* is a complex taxon that demonstrates remarkable genetic heterogeneity [4], [5]. Natural populations of *T. cruzi* undergo clonal evolution with rare events of genetic recombination [6]. However, hybrid lineages have been identified in natural *T. cruzi* populations [7], [8], [9], [10]. On the basis of a number of genetic and biochemical markers, the strains of *T. cruzi* have been divided into six discrete typing units (DTU) designed as TcI to VI [11].

Trypanosome genetic material is organized into small chromosomes, which are poorly condensed during cell division, precluding the use of conventional cytogenetic analysis. The *T. cruzi* karyotype is poorly defined; identification of each of the individual chromosomes has been problematic as many are of small-size or very similar. Therefore, the precise relationships between homologous chromosomes have yet to be determined. There is a significant variation in the size of chromosomes among strains [12], [13], [14], [15], [16], [17], and although the genome is generally diploid, the sizes of homologous chromosomes differ considerably [13], [14], [18], [19], [20]. Differences of up to 50% in the sizes of genetically equivalent chromosomes were detected in the karyotypes of various strains, suggesting that major chromosomal rearrangements occurred during the evolution of *T. cruzi*.

Studies based on flow cytometry, microfluorometry, chemical and renaturation kinetic analyses have demonstrated variation of up to 40% of the total DNA content among *T. cruzi* strains and clones [21], [22], [23], [24], [25], [26], [27]. The absolute amount of total DNA (nuclear + kinetoplast) varies from 0.12 to 0.33 pg per cell among various strains and clones isolated from the same strain [21], [22], [23], [25], [27]. The wide variation in genome size observed among eukaryotic species is more closely correlated with the amount of repetitive DNA than with the number of coding genes. In *T. cruzi* repeat sequences account for at least 50% [28]. The genome of *T. cruzi* was sequenced using a whole-genome sequencing approach from a hybrid strain (clone CL Brener) originated from genetic recombination of TcII and TcIII [8], [11], [28], [29]. Sequence strategy resulted in high sequence coverage from two parental haplotypes. However, high allelic variation and the repetitive nature of the genome have prevented the complete linear sequence of *T. cruzi* chromosomes being determined. Therefore, determining the full complement of chromosomes, identifying chromosome-specific markers and establishing syntenic and linkage genetic groups are important for defining the molecular karyotype and structure of *T. cruzi* chromosomes.

In this study, the genome organization of various *T. cruzi* strains was analyzed using genetic and computational approaches. The following questions were addressed: (1) What is the range of genome sizes and repetitive DNA contents (satellite DNA and retrotransposons) across strains from *T. cruzi* DTUs?; (2) What is the contribution of repetitive DNA to variability in genome size and chromosomal polymorphism?; (3) By analyzing large homologous chromosomal segments, what is the level of synteny among these strains? To address these questions genome sizes, repetitive DNA contents, and the numbers and sizes of chromosomes of nine strains from four DTUs including two clones from a hybrid strain, were determined and compared. The size distribution of syntenic blocks of clone CL Brener among the *T. cruzi* DTUs was examined. Two *T. cruzi* megabase chromosomes were compared with their counterparts in *T. brucei*; comparison between *T. cruzi* and *T. brucei* chromosomes could help the reconstruction of the ancestral trypanosome karyotype.

## Methods

### Ethics Statement

This study was carried out in strict accordance with the recommendations in the Guide for the Care and Use of Laboratory Animals of the National Institutes of Health. The protocol was approved by the Committee on the Ethics of Animal Experiments of the Federal University of Sao Paulo (Permit Number: CEP09555-07). All surgery was performed under sodium pentobarbital anesthesia, and all efforts were made to minimize suffering.

### Parasites

Nine isolates from the main lineages of *T. cruzi* [11] were used in this study. Three isolates belonged to group TcI (clone Dm28c, Tc1161 (José-IMT) isolate and G strain), two to the TcII group (clone Esmeraldo-cl3 and Y strain), one to the TcV group (clone SO3-cl5) and three to the TcVI group (CL strain, CL-strain derived clones CL Brener and CL 14). CL Brener was kindly provided by Dr. Bianca Zingales (IQ-USP), clone Dm28c by Dr. Samuel Goldenberg (ICC-Fiocruz), Y, G and CL strains and CL-strain derived clone CL 14 by Dr. Nobuko Yoshida (UNIFESP), clone SO3-cl5 by Dr. Marta de Lana (UFOP) and clone Esmeraldo-cl3 by Dr. Santuza Teixeira (UFMG). Tc1161 (José-IMT) was obtained from the Trypanosomatid Culture Collection (TryCC) of the Department of Parasitology, USP and was provided by Marta M. G. Teixeira (ICB-USP). Parasites were maintained by cyclic passage in mice and in axenic cultures at 28 °C in liver-infusion tryptose medium (LIT) containing 10% fetal calf serum.

### Synchronization of parasite cultures and flow cytometry

Epimastigotes were diluted to a final concentration of 3×10^6^ parasites per mL and maintained in the exponential growth phase for 24 h at 28°C. Hydroxyurea (20 mM) was added to the cultures, and after incubation for 24 h the number of parasites was determined using a Neubauer-counting chamber. Cells were washed with PBS and fixed with 50% methanol for 10 min at 0°C. After washing with PBS, parasites were resuspended in PBS (6×10^6^ cells/mL) containing 20 µg/mL propidium iodide and 16 µg/mL RNase and subjected to flow cytometry analysis in a custom-designed flow cytometer (Becton-Dickinson FACScalibur). Cultures without hydroxyurea (HU) were used as controls.

### Determination of total DNA content

Genome size of isolates from groups TcI (clone Dm28c, Tc1161 isolate and G strain), TcII (clone Esmeraldo-cl3 and Y strain), TcV (clone SO3-cl5) and TcVI (CL strain, CL-strain derived clones CL Brener and CL 14) were determined after several control experiments had been carried out. Epimastigotes of *T. cruzi* strains were arrested with HU and total DNA was isolated from 10^8^ cells as described previously [30]. DNA content was estimated using a fluorescent nucleic acid stain for dsDNA “Quant-iT™ dsDNA Assay Kit, High Sensitivity - 0.2-100 ng” (Invitrogen) as described. This kit provides accurate quantification, comparing DNA samples with pre-diluted DNA standards. Fluorescence intensity was read on a GENios Fluorometer (Magellan program) using 485 nm excitation and 535 nm emission wavelengths. Between three and seven independent assays were performed on each isolate and all experiments were carried out in triplicate. To assess the reliability of the assay using large DNA molecules, unbroken chromosomal DNA and sonic wave broken DNA were compared. Control experiments demonstrated that fluorophore incorporation was the same for both samples (data not shown). The next control experiment involved calculating the percentage yield obtained in DNA extraction experiments using a recovered radioactive experiment. *T. cruzi* chromosomal DNA was incubated overnight with 10 U endonuclease AscI in an appropriate restriction buffer at 37°C. After digestion, restriction fragments were labeled with 5 µCi [α-^32^P]dCTP, 0.02 mM dGTP and 5 U Klenow fragment in the same buffer at 65°C for 1 h (adapted protocol described by Cornillot et al., 2002) [31]. Known amounts of labeled DNA were added before total DNA extraction. Taking into account the percentage of radioactivity recovered, the real DNA mass in each *T. cruzi* isolate could be determined. Three independent assays were performed in triplicate (data not shown).

For statistical analysis, the one-way ANOVA test was performed using GraphPad InStat version 3.05 (GraphPad software, San Diego, CA). The statistical significance level was set at P<0.05. Data presented were the result of a minimum of three independent experiments and plotted as mean ± SD.

### Copy-number measurements

The copy numbers of repetitive sequences were determined approximately using dot-blot hybridization. DNA samples were denatured with NaOH (0.4 M) for 10 min, chilled on ice and diluted with an equal volume of 2 M ammonium acetate. DNA was quantified using an ultra-sensitive fluorescent nucleic acid stain for double-stranded DNA [“Quant-iT™ dsDNA Assay Kit, High Sensitivity - 0.2-100 ng” kit (Invitrogen)]. Various amounts of genomic DNA from *T. cruzi* strains (clones Dm28c, Esmeraldo-cl3, SO3-cl5 and CL Brener; Tc1161 isolate; G and Y strains) were applied to nylon membranes (Amersham) using a dot-blot apparatus (Bio-Rad). DNA was fixed by exposure to 150 mJ of UV radiation in a “GS Gene Linker™ UV chamber” (Bio-Rad).

Standard samples containing repetitive sequences DNA were loaded on the same filters to provide a standard scale. The following recombinant plasmids were used: clone F3.17, which carries part of the intergenic region from the L1Tc retrotransposon and part of reverse transcriptase (nt 1333 to 2021 of L1Tc, GenBank accession number X83098); clone F4.10, which carries 3.3 units of satellite DNA (195-bp repeats, GenBank accession number AY520076) and pUC18 as a background control. Filters were hybridized in exactly the same way as the chromoblots. After autoradiographic exposure, the amount of ^32^P in each spot was determined by liquid scintillation counting. The amount of probe sequence in the trypanosome DNA samples was estimated from a graph of the counts present in the spots of each repetitive sequence. Copy numbers of repetitive sequences in the various *T. cruzi* genomes were calculated taking into consideration the genome size determined in this study. The pUC18 control was used to normalize experiment data.

For statistical analysis, a one-way ANOVA test was performed using GraphPad InStat version 3.05 (GraphPad software, San Diego, CA). The statistical significance level was set at P<0.05. Data presented were the result of at least three independent experiments and plotted as mean ± SD.

### Separation of *T. cruzi* chromosomal DNA by pulsed-field gel electrophoresis (PFGE)

Epimastigotes from *T. cruzi* were grown to late logarithmic phase. Cells were collected in PBS and mixed with an equal volume of 1% low-melting point agarose. Approximately 1×10^7^ cells (100 µL) were used for each gel plug; these were incubated in a solution containing 0.5 M EDTA (pH 8.0), 1% sodium lauryl sarcosinate (Sarkosyl) and 1 mg/mL proteinase K at 50°C for 48 h, and stored at 4°C in 0.5 M EDTA (pH 8.0). Chromosomal bands were separated on agarose gels using a Gene Navigator System (Amersham Pharmacia Biotech, NJ, USA) and a hexagonal electrode array. PFGE was carried out using 1.2% agarose gels in 0.5X TBE (45 mM Tris; 45 mM boric acid; 1 mM EDTA, pH 8.3) at 13°C for 132 h as previously described [18]. Gels were stained with ethidium bromide (0.5 µg/mL) and photographed. DNA samples were incubated with 0.25 M HCl for 45 min, denatured with 0.5 M NaOH/1 M NaCl for 20 min, neutralized with 1 M Tris-base/0.5 M NaCl for 20 min and transferred to nylon membranes in 20X SSC (1X SSC = 0.15 M NaCl and 0.015 M sodium citrate). The membranes were hybridized as described below.

### Hybridization

Membranes were pre-hybridized in a solution containing 50% formamide/5X SSC/5X Denhardt's solution (Invitrogen)/0.1 mg/mL salmon sperm DNA/ 0.1 mg/mL tRNA at 42°C for 1 h and hybridized overnight at 42°C with ^32^P-labeled probes. Following hybridization, membranes were subjected to two washes (30 min each at 42 °C) in 2X SSC containing 0.1% SDS and 0.1% sodium pyrophosphate and two additional washes at 56 °C in 0.1X SSC containing 0.1% SDS and 0.1% sodium pyrophosphate. They were then exposed to X-ray film. The gene identification and the accession number of each marker used as probe are indicated in Table S4.

### Bioinformatic analysis

*T. cruzi* contigs and scaffolds were assembled into 41 platforms named chromosomes (TcChr)[32]. The chromosome-sized scaffolds assigned to the Esmeraldo and non-Esmeraldo haplotypes were designated S and P, respectively. The *T. cruzi* clone CL Brener (TcChr) and *T. brucei* (Tb) chromosomes used in this study were obtained from EuPathDB Project (http://tritrypdb.org/tritrypdb). Whole genome alignments between TcChr P and S were performed using tblastx algorithm [33] and implemented through big_blast.pl script (from Sanger Institute) that was modified by Jeronimo Ruiz. TcChr chromosomes were also used in similarity searches using tblastx algorithm against the *T. brucei* genomic sequence.

A locally compiled database (DB) of *T. cruzi* sequences was built by parsing sequences from GenBank. Chromosome-specific markers from this database were used as anchors in similarity searches. Similarity searches against this locally compiled DB were carried out using the BLAST and FASTA program package algorithms [34], [35]. The annotation and graphical output of chromosome-specific markers were obtained using the Artemis Comparison Tool [36] (http://www.sanger. ac.uk/resources/software/act).

## Results

We selected nine strains from the four major *T. cruzi* lineages [TcI (DTU I), TcII (DTU IIb), TcV (DTU IId) and TcVI (DTU IIe)] that are very well characterized in terms of epidemiological, biological and pathological features [4], [8], [11], [21], [37], [38], [39], [40]. The isolates of DTUs I, II, V and VI predominate in endemic areas and are responsible for most cases of human Chagas' disease in Central America (mostly TcI) and South America (mostly TcII). Isolates of DTU I are the most widespread isolates of *T. cruzi* circulating in sylvatic cycles (in all Latin America) and domestic cycles (in Central America, Colombia and Venezuela). Characterization of a large number of isolates evidenced important genetic population diversity within DTU I [39], [41], [42]. For these reasons, three isolates from TcI diverging in host and geographic origin were included in this study.

### Fluorescent nucleic acid stain for double-stranded DNA accurately estimates genome sizes of ***T. cruzi*** isolates

The genome size, repetitive DNA content and karyotype of nine different isolates from the main lineages of *T. cruzi* were estimated and compared. Clone CL Brener (TcVI) was chosen for this study as it is the reference strain for the genome sequencing project. The ability of the fluorescent dye to estimate the genome size accurately in three isolates from group TcI (clone Dm28c, Tc1161 isolate and G strain), two isolates from group TcII (clone Esmeraldo-cl3 and Y strain), one isolate from group TcV (clone SO3-cl5) and three isolates from group TcVI (CL strain and CL-derived clones CL Brener and CL 14) was assessed. The analysis was performed using parasites arrested with HU, which inactivates ribonucleoside diphosphate reductase, thereby preventing cells from leaving the G1/S phase of the cell cycle. Non-treated trypanosomes presented with a profile comprising two peaks representing cells with 2C and 4C DNA contents, respectively (Fig. S1). After 24 h incubation with HU, the proportion of 2C cells increased at least 1.7-fold compared with the corresponding 4C cells, indicating that epimastigotes were arrested in the G1 phase (Fig. S1). These results are in agreement with those obtained by Elias et al. [43]. Cells that were post-S phase at the time of HU addition would have progressed through the cell cycle and re-entered G1.

Epimastigote cells in the G1-phase of the cell cycle were used to estimate DNA content by quantifying the dsDNA. Control experiments were performed using well-established haploid and diploid *S. cerevisiae* lineages. The estimated nuclear DNA contents of haploid and diploid yeast lineages were 0.013860±0.001989 and 0.026348 ± 0.006182 pg/cell, respectively. Table 1 summarizes the estimates of total DNA content per cell (nucleus and kDNA) for *T. cruzi* isolates. Values refer to the diploid content, assuming that *T. cruzi* is essentially diploid [28]. Between three and seven independent assays were performed on each isolate. Variance was analyzed (ANOVA test) to detect significant differences among the isolates (Table S1). The mean total DNA contents of parasites from groups II (Esmeraldo-cl3 and Y strain), V (clone SO3-cl5) and VI (CL strain, CL-strain derived clones CL Brener and CL 14) were higher than isolates from group TcI (G strain and clone Dm28c). Although Tc1161 belongs to the TcI group [11], [44], its genome is larger than other isolates from this group. These differences were significant when assessed using hierarchical ANOVA (Table S1). There was no significant difference (P>0.05) among parasites from *T. cruzi* groups II (Esmeraldo-cl3 and Y strain), V (clone SO3-cl5) and VI (CL-strain derived clones CL Brener and CL 14). Significant differences (P<0.05) were demonstrated between clone Esmeraldo-cl3 and CL strain, and CL strain and clone CL 14.

**Table 1:** Total DNA content and genome size of various *T. cruzi* isolates.

Isolate	Group	Total DNA content* (ρg)			Genome size** (Mb)	Nuclear genome*** (Mb)
G	I	0.122270	±	0.026692	112.17	90
Dm28c	I	0.121429	±	0.031253	111.40	89
Tc1161	I	0.157183	±	0.031282	144.20	115
Y	II	0.171660	±	0.044942	157.49	126
Esmeraldo	II	0.155235	±	0.029427	142.42	114
SO3-cl5	V	0.183778	±	0.024398	168.60	135
CL	VI	0.191118	±	0.038978	175.34	140
CL14	VI	0.152632	±	0.015553	140.03	112
CL Brener	VI	0.165813	±	0.031826	152.12	122

*Absolute DNA mass (ρg) by means of fluorescent nucleic acid stain assay per epimastigote cell. Values are the means ± SD of three to seven independent assays performed in triplicate.

**Estimate of genome size including nuclear and kDNA considering 1 base pair as 1.09×10^−9^ ρg.

*** Nuclear genome size was estimated assuming that kDNA accounts for 20% of total DNA [45].

Assuming that kDNA accounts for 20% of the parasite's total DNA [21], [45], the nuclear genome size was determined for each isolate (Table 1). The nuclear genome of clone CL Brener was estimated to be 122 Mb, although a previous study demonstrated that the genome size was 106.4–110.7 Mb [28]. It is likely that the discrepancy (11 Mb) between the data presented herein and that from the sequencing genome project represents extra repetitive sequences in non-sequenced gaps in repeated regions such as nucleolar organizing regions, spliced leader tandem repeats and individual reads that were not assembled in contig sequences [46]. From the data presented in this study, the nuclear genome is larger than previously thought and its size varies up to 1.57-fold between strains (e.g. CL *vs* Dm28c) and 1.25-fold within isolates from the same strain (e.g. CL strain *vs* clone CL14). TcI isolates have, on average, smaller nuclear genomes (89.5 Mb, G and Dm28c) than TcII, TcV and TcVI isolates (125 Mb). These results concurred with previous reports [18], [19] demonstrating that TcI isolates have smaller genomes than TcII isolates. The only exception to this rule was the isolate Tc1161, whose genome size (115 Mb) is comparable to TcII, TcV and TcVI, and higher (P<0.05) than other TcI isolates, G strain and clone Dm28c (89.5 Mb).

### Copy number of repetitive sequences

Genome size differences could be attributed to the amplification and deletion of various repeated DNA sequences including retrotransposons and satellite DNA. To investigate the influence of repetitive DNA fractions on the genome size of *T. cruzi* isolates, the copy numbers of a high-repetitive sequence (195-bp satellite DNA element) and a middle-copy number non-LTR retrotransposon (L1Tc) were estimated. The copy number of the repetitive sequences was estimated using dot-blot hybridization and known amounts of *T. cruzi* genomic DNA and recombinant plasmids containing the repetitive element. The hybridization signal intensity was quantified by measuring the amount of ^32^P in each spot by liquid scintillation counting. The copy numbers of satellite DNA per cell were estimated to be 35,474 and 29,886 in Y and Esmeraldo-cl3, respectively (TcII isolates); 29,459 and 27,890 copies in the SO3-cl5 and CL Brener, respectively (TcV and TcVI isolates); and from 9,247 to 12,382 in TcI isolates (G, Dm28c and Tc1161) (Table 2). Satellite DNA is 2.3 to 3.8 times more abundant in TcII, TcV and TcVI isolates than in TcI isolates. Variance analysis (ANOVA test) demonstrated that the trend was significant (P<0.001). As shown in Tables 2 and S2A, the Y strain contains more satellite DNA and this variance is significant when compared with other isolates (P<0.001), while no significant difference (P>0.05) was found among Esmeraldo-cl3 (TcII), clone SO3-cl5 (TcV) and clone CL Brener (TcVI). There was no significant difference (P>0.05) among the TcI isolates (clone Dm28c, Tc1161 isolate and G strain) (Table S2A). These data are in agreement with a previous report that suggested repetitive sequences are less abundant in TcI genomes than TcII, V and VI genomes [16], [17].

**Table 2:** Copy number of Satellite DNA and L1Tc retrotransposon in various *T. cruzi* isolates.

Isolate	Group	Satellite DNA*			L1Tc*		
G	I	9341	±	766	323	±	26
Dm28c	I	12382	±	1261	586	±	33
Tc1161	I	9247	±	1077	720	±	15
Y	II	35474	±	2231	561	±	25
Esmeraldo	II	29886	±	1526	398	±	17
SO3-cl5	V	29459	±	3053	726	±	33
CL Brener	VI	27890	±	1878	635	±	27

*Values are the means ± SD of three independent assays performed in duplicate.

L1Tc presented 1.2 to 2.2-fold variation in copy number among the various isolates. Significant differences were evident (P<0.001) between of the majority of the isolates (Table S2B). However, no significant difference (P>0.05) was demonstrated between Dm28c and Y, Dm28c and CL Brener, or Tc1161 and SO3-cl5. L1Tc is more abundant in Tc1161 and SO3-cl5 genomes and less abundant in the G strain and clone Esmeraldo-cl3. There was no association between high numbers of this repetitive sequence and TcII, V and VI groups.

Satellite DNA and L1Tc fractions account for 4.47% (5.44 Mb) and 2.6% (3.18 Mb), respectively, of the genome of clone CL Brener. This is in agreement with an estimate of abundance of satellite DNA (5.13%) in clone CL Brener [46] using individual reads rather than assembled contig sequences generated by the *T. cruzi* sequencing consortium [28]. Satellite DNA and L1Tc fractions comprised 8.62 Mb or 7.1% of the nuclear genome of CL Brener. The content of a *T. cruzi* species-specific sequence (TcTREZO) in the genomes of CL Brener, G, Dm28c and Tc1161 was determined. TcTREZO is a site-specific element composed of three sub-regions that have sequence similarity with other *T. cruzi* sequences [47]. The copy number of TcTREZO-related sequences was estimated to be 3,998 elements per cell in CL Brener and 1,693; 1,546; 1,593 copies in G, Dm28c and Tc1161, respectively. TcTREZO is 2.5 times more abundant in CL Brener than TcI isolates. Previously, we used the RepeatMasker script to estimate the number of copies of TcTREZO in the *T. cruzi* database [47]. Using the 1,573-bp sequence of TcTREZO (AF508945) formatted as a custom library, we identified 173 copies of the complete element per haploid genome. Taken together, these results suggest that the majority of TcTREZO tandem sequences were not incorporated into the CL Brener assembled contig sequences. Furthermore, most of TcTREZO sequences are truncated (94%).

Our results are in agreement with those from Sylvio X10/1 genome project, a TcI isolate which genome was fully sequenced recently [48]. By comparison repetitive sequences content between Sylvio X10/1 and CL Brener, Franzen et al. did not find significant difference in LTR/LINE copy number and low number of satellite DNA comparing to CL Brener genome. The Sylvio X10/1 genome size was estimated to be 88 Mb, similar to TcI genomes studied herein (G strain and Dm28c).Furthermore, they confirmed that multigenic families (e.g. MASP, mucin, DGF-1, GP63 and RHS gene families) are less abundant in Sylvio X10/1 and underlie genome size difference between these two genomes fully sequenced (Sylvio X10/1 and CL Brener) [48].

### Karyotype polymorphism

**Figure 1:**
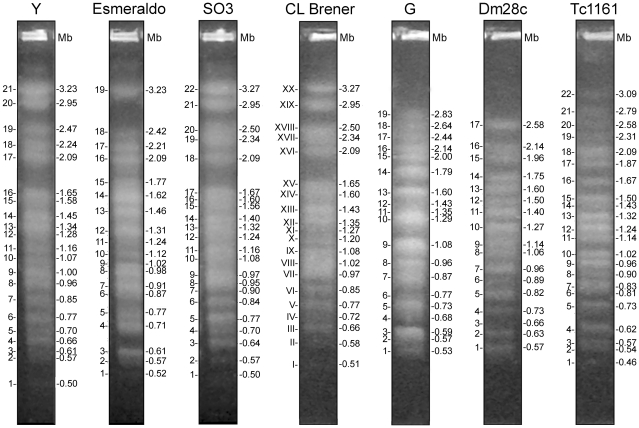
Karyotype polymorphism in representative isolates of *T. cruzi* lineages. Chromosomal bands were separated by Pulsed Field Gel Electrophoresis (PFGE) and stained with ethidium bromide. The chromosomal bands of clone CL Brener were numbered using Roman numerals (I – XX) and Dm28c with Arabic numerals as described previously [17], [18]. The bands of others isolates were numbered using Arabic numerals starting from the smallest band, as the reference determined for clone CL Brener [18]. The following isolates were used: Y strain and clone Esmeraldo-cl3 from lineage TcII; clone SO3-cl5 from TcV; CL Brener from TcVI; clone Dm28c, G strain and Tc1161 (José-IMT) isolate from TcI. Numbers of chromosomal bands and their sizes are indicated at left and at right of each strip, respectively.

Chromosomal bands were separated by PFGE and stained with ethidium bromide (Fig. 1). Herein, chromosomal bands are defined as those bands separated by PFGE and visible after staining with ethidium bromide. The distribution of ethidium bromide fluorescence was not the same for all chromosomal bands, indicating that co-migrating chromosomes are not necessarily homologous. A chromosome or homologue is a single DNA molecule. The chromosomal bands of CL Brener were numbered using Roman numerals (I–XX) and Dm28c with Arabic numerals as previously described [17], [18]. The bands of other isolates were numbered using Arabic numerals, starting with the smallest band (Fig. 1). The karyotype patterns are homogeneous within TcII, V and VI isolates. It is composed of 19–22 bands with sizes ranging from 3.27 to 0.5 Mb, and they can be easily differentiated from those of TcI isolates (G strain and clone Dm28c). As previously reported [16], [17], the karyotypes of TcII, V and VI are generally different from TcI isolates (G strain and clone Dm28c). Nineteen bands ranging from 0.53 to 2.83 Mb in G strain, and 17 bands ranging from 0.57 to 2.58 Mb in clone Dm28c, were identified. The size and number of chromosomal bands in the G strain and clone Dm28c were smaller than those identified in TcII, V and VI isolates. The Tc1161 genome displayed 22 chromosomal bands ranging from 0.46 to 3.09 Mb. The chromosomal bands were larger in size and number than TcI isolates. It is interesting to note that although the isolate Tc1161 belongs to TcI, its chromosomal pattern is more similar to that of the TcII, V and VI isolates. This is in agreement with the estimated value of the Tc1161 genome size, which is higher than that of other TcI isolates.

Hybridization of chromosomal bands with repetitive sequences (satellite DNA, L1Tc) (Fig. S2) confirmed the copy number estimates reported above. The satellite DNA strongly hybridized to 9–12 bands of TcII, V and VI isolates, and with lesser intensity to 7–10 bands in TcI isolates (Fig. S2 and Table S3A). It hybridized with approximately 50% of chromosomal bands but predominantly larger ones. The intensity of the hybridization signal was markedly stronger in the chromosomal bands of TcII, V and VI, suggesting the presence of a higher DNA satellite copy number per cell in these isolates. This is in agreement with the dot-blot estimates that demonstrated that the satellite DNA was 2.3 to 3.8 times more abundant in TcII, V and VI isolates than in TcI isolates. The retrotransposon L1Tc was distributed evenly in almost all chromosomal bands of TcI, II, V and VI isolates, with the exception of clone Esmeraldo-cl3 (Fig. S2 and Table S3B). Using the intensity of signal hybridization, there was a high concentration of L1Tc in the Y, SO3-cl5, CL Brener and Tc1161 genomes and it was distributed among many chromosomal bands. L1Tc was concentrated in bands ranging from 1.06 to 1.50 Mb of clone Dm28c. As expected from the copy number of estimates (Table 2), L1Tc was less abundant in G strain and clone Esmeraldo-cl3. The distribution of satellite DNA and L1Tc in the chromosomal bands was consistent with its copy numbers in the different isolates.

### Large-scale synteny in ***T. cruzi*** lineages

The concept of synteny, molecular markers shared between chromosomes and organized in the same order, was used to define regions of chromosomal homology. To examine the level of synteny among the TcI, II, V and VI isolates, genetic markers previously mapped to the chromosomal bands XVI (2.09 Mb) and XX (3.27 Mb) of clone CL Brener were used. Physical maps of chromosomes XVI [49] and XX [50] have been constructed using YAC clones and hybridization with chromosome-specific markers. Table S4 lists the chromosome specific markers used as probes in the hybridization experiments, and includes the gene identification, the accession number and the chromosomal bands identified in clone CL Brener. Here, the newly generated sequence assemblies of clone CL Brener identified by the EuPathDB Project (TrypDB: http://tritrypdb.org) [32] were integrated into the physical map of megabase chromosomes XVI and XX of clone CL Brener.

**Figure 2:**
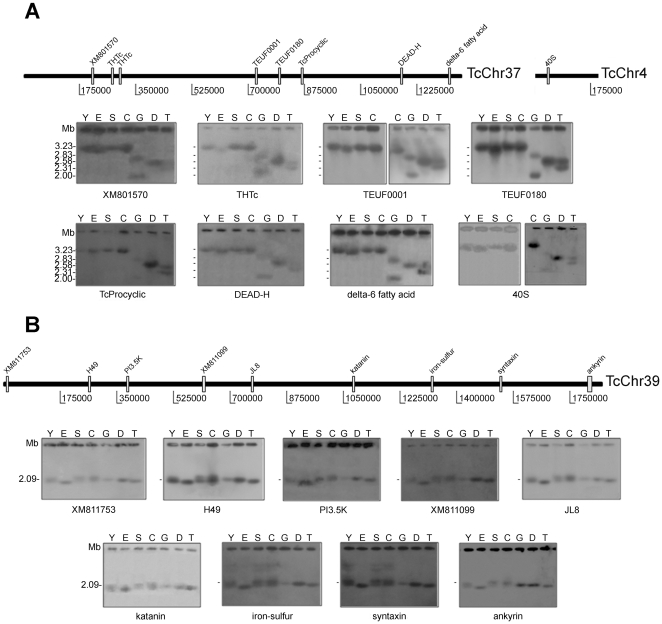
Conservation of large syntenic regions in the genome of *T. cruzi* lineages. Linkage groups from TcChr37 and TcChr4 (**Panel A**) and TcChr39 (**Panel B**) were mapped on chromosomal bands. Specific markers from *in silico* assembled *T. cruzi* chromosomes (TcChr) were hybridized with chromosomal bands separated by PFGE. The positions of markers used as probes are indicated in the diagrammatic representation of *in silico* assembled chromosomes (TcChr37 and 4, and TcChr39).The isolates are: Y and clone Esmeraldo-cl3 (E) from lineage TcII; clone SO3-cl5 (S) from TcV; clone CL Brener (C) from TcVI; clone Dm28c (D), G strain and Tc1161 (José-IMT) isolate (T) from TcI. Markers from TcChr37: XM_801570, THTc, TEUF0001, TEUF0180, TcProcyclic, DEAD-H and delta-6. The marker 40S is located in TcChr4. Markers from TcChr39: XM_811753, H49, PI3,5K, XM_811099, JL8, katanin, iron-sulfur, syntaxin and ankyrin. The gene identification and GenBank accession number of each marker are indicated in Table S4.

**Figure 3:**
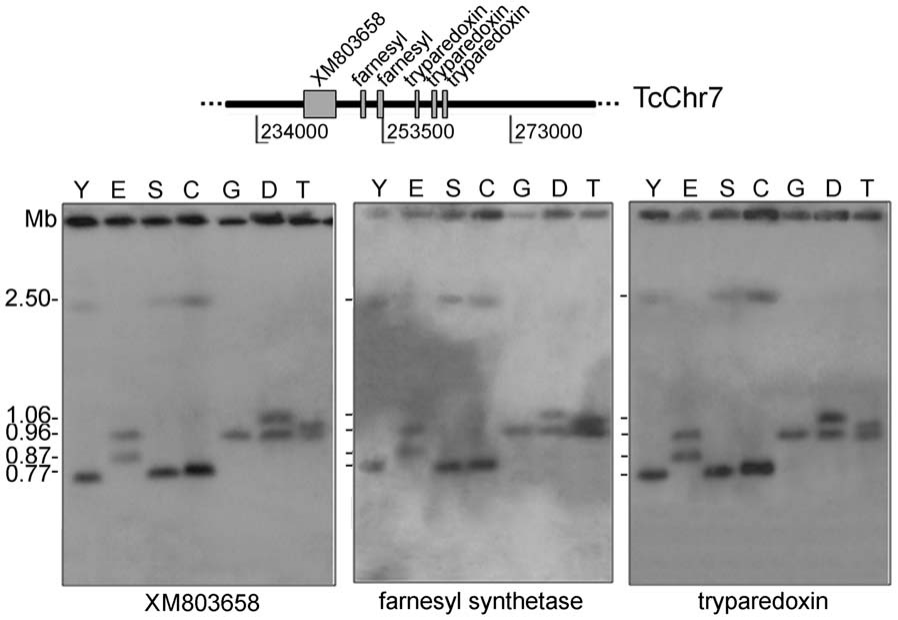
Segmental duplication on a 2.5 Mb chromosomal band in the Y strain and clones SO3-cl5 and CL Brener. A single segment in TcChr7 comprising the coding sequences XM_803658, farnesyl synthetase and tryparedoxin peroxidase corresponds to a gene segmental duplication on a 2.50 Mb chromosomal band in the Y strain and clones SO3-cl5 and CL Brener. The positions of markers used as probes are indicated in the diagrammatic representation of *in silico* assembled chromosome TcChr7. The isolates are: Y and clone Esmeraldo-cl3 (E) from lineage TcII; clone SO3-cl5 (S) from TcV; CL Brener (C) from TcVI; clone Dm28c (D), G strain and Tc1161 (José-IMT) isolate (T) from TcI. Markers from TcChr7: XM_803658, farnesyl synthetase and tryparedoxin peroxidase. The gene identification and GenBank accession number of each marker are indicated in Table S4.

Recently, *T. cruzi* contigs and scaffolds were assembled into 41 platforms, tentatively named as chromosomes (TcChr) [32]. This designation is not accurate as some of these chromosomes could be part of a single chromosome. For this reason, they are referred to as chromosome-sized scaffolds in the present study. They were assigned to the Esmeraldo and the non-Esmeraldo haplotypes and designated S and P, respectively [33]. The chromosome-sized scaffolds were assigned to the chromosomal bands of CL Brener separated by PFGE. The assignments obtained were: chromosomes TcChr37-P and S, and TcChr4-P and S to the electrophoretic band XX (3.27 Mb) (Fig. 2A); TcChr39-P and S to the electrophoretic band XVI (2.09 Mb) (Fig. 2B); TcChr7-P and S to the electrophoretic bands XVIII (2.5 Mb) and V (0.77 Mb) (Fig. 3).

The chromosomal band XX-specific markers delta-6-fatty acid desaturase, hexose transporter, TEUF0180 (85 kDa HSP), procyclic form surface glycoprotein and hypothetical protein XM_801570 were assigned to the chromosome-sized scaffolds TcChr37-P and TcChr37-S, while TEUF0001 (Histone H2B) and XM_814424 (ATP-dependent DEAD/H RNA helicase) were assigned to the TcChr37-P and TcChr37-S, respectively (Fig. 2A). The delta-6-fatty acid desaturase gene was located at a distance of approx 1.11 Mb from the hypothetical protein gene XM_801570 (see Fig. 2A). The 40S ribosomal protein S24E was assigned to the chromosome-sized scaffold TcChr4-P. All markers identified in TcChr37 hybridized to one chromosomal band in the TcII (3.23 Mb), TcV and VI isolates (3.27 Mb) and in the clone Dm28c (2.58 Mb), and with two distinct bands in TcI isolates (2.00 and 2.83 Mb in G strain; 2.31 and 2.58 Mb in Tc1161). The same type of pattern was observed for the 40S ribosomal protein S24E marker (Fig. 2A, Table S4), suggesting that TcChr37 and TcChr4 scaffolds are part of the same chromosome. The results confirm the linkage of these markers on the non-Esmeraldo-like “P” and the Esmeraldo-like “S” chromosomes, and indicate that the syntenic block was conserved in TcI, II, V and VI isolates. The co-localization of these markers with two chromosomal bands in TcI isolates (2.00 and 2.83 Mb in G strain; 2.31 and 2.58 Mb in Tc1161) could be explained by the existence of two different-sized homologous chromosomes or by the occurrence of a large duplication event comprising the 1.1 Mb regions of two non-homologous chromosomes.

The chromosomal band XVI-specific markers phosphatidylinositol (3,5) kinase, H49, JL8, katanin, iron-sulfur cluster assembly protein, syntaxin and ankyrin were assigned to the chromosome-sized scaffolds TcChr39-P and TcChr39-S, while the hypothetical protein XM_811099 was assigned to the TcChr39-S, and hypothetical protein XM_811753 to the TcChr39-P. TcChr39-P and TcChr39-S are approximately 1.85 Mb in length. The XM_811753 was located approx 1.79 Mb from ankyrin (Fig. 2B). These markers hybridized with two chromosomal bands in the Y strain (2.09 and 2.24 Mb), CL Brener and SO3-cl5 (2.09 and 2.34 Mb), and with a chromosomal band (2.09 or 2.14 Mb) in Esmeraldo-cl3 and TcI isolates (Fig. 2B). The two chromosomal bands identified in hybrids and Y could be different-sized chromosomes, as demonstrated in clone CL Brener [49]. The results confirm that this large syntenic block is conserved in TcI, TcII, TcV and TcVI isolates.

Despite strong chromosomal conservation, some markers are found only in one haplotype (TcChr-P or TcChr-S) of clone CL Brener. They are not necessarily specific-haplotype markers because the corresponding region of each marker is interrupted by sequence gaps designated as an N-rich region (nucleotides not determined). There are 56 to 75 N-rich regions in TcChr37-P, TcChr37-S, TcChr39-P and TcChr39-S, comprising 10.4% to 20.5% of these haplotypes. This could explain the presence of 40S ribosomal protein S24E in TcChr4-P and the many mismatches identified in the merging of syntenic regions from Esmeraldo and non-Esmeraldo haplotypes [32].

A linkage group assigned to the chromosome-sized scaffolds TcChr7-P and TcChr7-S was analyzed. These markers hybridized in two chromosomal bands (0.96 and 1.06 Mb in clone Dm28c and 0.96 and 1.02 Mb in Tc1161) of TcI isolates, with the exception of the G strain in which only the 0.96 Mb band was detected (Fig. 3 and Table S4). Two chromosomal bands (0.87 and 0.98 Mb) were identified in the clone Esmeraldo-cl3 of TcII. In CL Brener, SO3-cl5 and Y, the probes strongly hybridized to a 0.77 Mb-band and weakly to a 2.5 Mb-band. These results suggest that the 0.77 Mb band harbors two homologous chromosomes of the same size. Hybridization of TcChr7 markers with the 2.5 Mb-band could be the result of spontaneous duplication of a DNA segment comprising the coding sequences XM_803658, farnesyl synthetase and tryparedoxin peroxidase, followed by translocation to the 2.5 Mb chromosomal band. The fragment comprising from XM_803658 to the third copy of tryparedoxin peroxidase gene is approx 20.2 kb (Fig. 3).

### Chromosome architecture and comparison with *T. brucei* chromosomes

**Figure 4:**
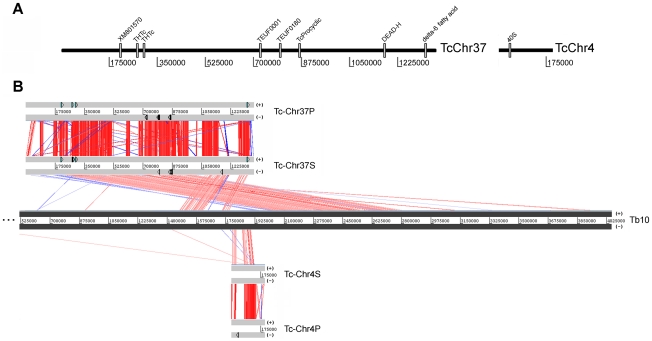
Alignment among homologous regions of *T. cruzi in silico* assembled chromosomes TcChr37, TcChr4 and *T. brucei* chromosome Tb10. Panel A) Schematic representation of chromosome-specific markers from the chromosomal band XX (clone CL Brener) in the assembly sequence of chromosome TcChr37 and TcChr4 (clone CL Brener). These markers were used as probes in chromoblot hybridizations. Panel B) Comparison among TcChr37 (“P” chromosome assigned to the non-Esmeraldo haplotype and “S” to the Esmeraldo haplotype) and TcChr4 (“P” and “S”) with chromosome Tb10 of *T. brucei*. Homologous genes are connected by colored lines. The matches and reverse matches are represented in red and in blue, respectively. The beginning of TcChr37 (1 Mb) aligned to a chromosomal segment located in the middle of Tb10 (positions 2 Mb to 2.8 Mb). The end of TcChr37 (350 kb) shows similarity with the portion included between 1.43 Mb to 1.75 Mb of Tb10. The TcChr4 made up the middle of Tb10, where there is a large chromosome inversion. Grey blocks represent each chromosome. Chromosome-specific markers are drawn in sense (+) and antisense (-) strands.

*T. brucei* and *T. cruzi* exhibit striking conservation of gene order [51], [52]. *T. brucei* chromosomes provide a structural basis for studying *T. cruzi* chromosome organization [32]. The data presented herein demonstrated that TcChr37 and TcChr4 specific markers map to the chromosomal band XX (3.27 Mb) of clone CL Brener. Correlations between *T. cruzi* and *T. brucei* chromosomes were accomplished using sequences of large chromosomal fragments from each species. TcChr37 was identified as being homologous to *T. brucei* chromosome 10 (Tb10) (Fig. 4). The comparison between these chromosomes identified a large segment inversion involving chromosome TcChr37. The initial segment of TcChr37 (1 Mb) corresponds to a region from 2.0 to 2.8 Mb of Tb10, while the ending segment of TcChr37 (350 kb) is similar to a region of 1.43 to 1.75 Mb of Tb 10 (Fig. 4). Furthermore, the region located between the two chromosome inversion segments in Tb10 corresponds to TcChr4. Although there was no assembling between these chromosome-sized scaffolds in the database, they belong to the same chromosome. On the basis of their gene content, telomeric sequences were identified at one end of TcChr37 and TcChr4 suggesting that they are located at the extremities of chromosome XX (see Fig. S3). The telomeric region is located at the start of TcChr37 and at the end of TcChr4, both in the middle of Tb10, suggesting there was a real inversion process in this *T. cruzi* chromosome (Fig. S3). The complete sequences of chromosome-sized scaffolds TcChr37 and TcChr4 comprise 1,361,061 and 200,400 bp, respectively which covers 47.75% of the entire chromosome XX (3.27 Mb). The difference in size can be explained by other TcChr not allocated to chromosomal band XX or by the highly repetitive nature of the genome, where the numerous repetitive regions were collapsed and/or misassembled.

**Figure 5:**
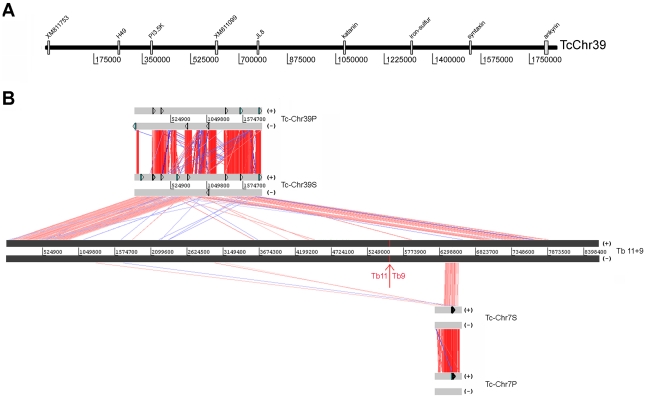
Alignment among homologous genomic regions of *T. cruzi in silico* assembled chromosomes TcChr39 and TcChr7, and *T. brucei* chromosomes Tb11 and Tb9. Panel A) Schematic representation of chromosome-specific markers from the chromosomal band XVI (clone CL Brener) in the assembly sequence of chromosome TcChr39 (clone CL Brener). These markers were used as probes in chromoblot hybridizations. Panel B) Comparison among TcChr39 (“P” chromosome assigned to the non-Esmeraldo haplotype and “S” to the Esmeraldo haplotype) and TcChr7 (“P” and “S”) with chromosomes Tb11 and Tb9 of *T. brucei*. For ease of viewing, chromosomes Tb11 and Tb9 were joined together and drawn in the same line. The limit between Tb11 and Tb9 is shown by a red arrow. Grey blocks represent each chromosome. Homologous genes are connected by colored lines. . The matches and reverse matches are represented in red and in blue, respectively. The beginning of TcChr39 (821 kb) was aligned to the initial portion of Tb11. The end of the TcChr39 (1 Mb) shows similarity with Tb9 end (positions 1.8 Mb to 2.8 Mb). TcChr7 constituted the beginning of Tb9 (390 kb region between positions 1.1-1.5 Mb). Chromosome-specific markers are drawn in sense (+) and antisense (-) strands.

The complete sequence of chromosome-sized scaffold TcChr39 comprises 1,849,755 bp. TcChr39 was assigned to two different *T. brucei* chromosomes: Tb9 and Tb11 (Fig. 5). The initial region of TcChr39 (821 kb) is homologous to a region of the same size located in one extremity of Tb11. The ending part (1 Mb) of TcChr39 is similar to Tb9 of *T. brucei* (position 1.8 to 2.8 Mb of Tb9). These two TcChr39 fragments, homologous to different *T. brucei* chromosomes, are separated by a short region (nt 877,707 to 883,237) corresponding to the *T. cruzi* specific sequences VIPER and C6 interspersed DNA elements. These sequences are generally located in synteny breakpoints [51], [52]. The presence of telomeric sequences at both ends of TcChr39 suggests that it represents the complete sequence. The length of 1,849,755 bp compares well with the PFGE-based estimate (mean of several determinations) of 2.09 Mb (see Fig. 2). The complete sequence of chromosome-sized scaffold TcChr39 includes 72–75 gaps which have been indicated in the TriTrypDB as blocks of 100 to 197,373 nt. The total size of these gaps is estimated as 192,815 bp in TcChr39-S and 261,904 bp in TcChr39-P, and they could be composed of repetitive sequences.

Using information from genome projects, the sequences of TcChr7 were compared with *T. brucei* chromosomes (see Fig. 5). TcChr7 is similar to the initial region of Tb9 (390 kb segment located between 1.1–1.5 Mb of Tb9), while the final region of Tb9 corresponds to TcChr39 (segment located between 1.8–2.8 Mb of Tb9). TcChr7 and TcChr39-specific markers hybridized with different chromosomal bands in CL Brener, suggesting that they are not located in the same chromosome (Figs. 2B and 3).

**Figure 6:**
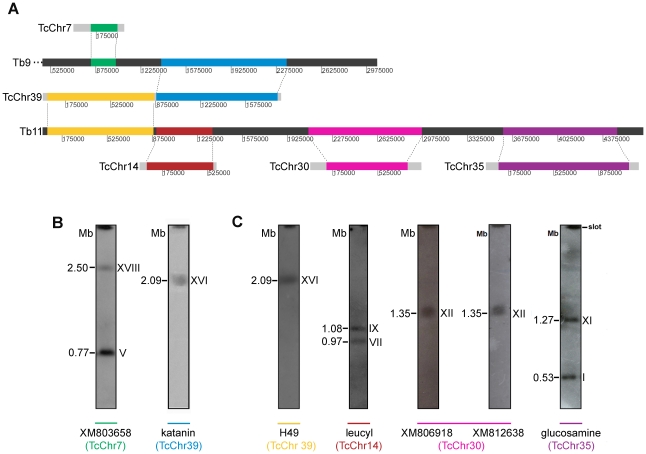
Split and fusion events among *T. cruzi* and *T. brucei* chromosomes. **Panel A**) Comparison among homologous regions of *T. cruzi* chromosomes TcChr39, TcChr7, TcChr14, TcChr30 and TcChr35 and *T. brucei* chromosomes Tb11 and Tb9. The homologous regions of TcChr7 (green box) and the end of TcChr39 (blue box) comprise the beginning and the end of Tb9, respectively. The beginning of TcChr39 and the entire *T. cruzi* chromosomes TcChr14, TcChr30 and TcChr35 were assigned to different regions of Tb11. Soft and dark grey blocks represent *T. cruzi* and *T. brucei* chromosomes, respectively. **Panel B**) Markers XM_803658 and katanin located in *T. cruzi* homologous regions to Tb9 were hybridized with chromosomal bands of clone CL Brener. **Panel C**) Markers located in *T. cruzi* homologous regions to Tb11 were hybridized with chromosomal bands of clone CL Brener. The probes were the following: H49, leucyl, XM_806918, XM_812638 and glucosamine. The gene identification and the accession number of each marker are indicated in Table S4.

To understand the complexity of fusion/split fragment processes between *T. cruzi* and *T. brucei* further, the predicted organization of each chromosome was associated by experimental Southern blot hybridization with the *T. cruzi* chromosomal bands separated by PFGE, and chromosome-specific genes were used as probes (Fig. 6). *T. brucei* chromosome Tb9 is syntenic to the distal ends of *T. cruzi* TcChr39 (blue block) and TcChr7 (green block) (Fig. 6A). However, Tb11 shares syntenic regions with the initial region of TcChr39 (yellow block) and the whole chromosomes TcChr14 (brown block), TcChr30 (pink block) and TcChr35 (purple block) (Fig. 6A). Hybridization of markers from *in silico* chromosomes (TcChr) to chromosomal bands of CL Brener is presented in Fig. 6B and 6C. The marker TcChr39 (H49) mapped to the chromosomal band XVI, the marker located in TcChr14 (leucyl tRNA synthetase) mapped to the chromosomal bands VII and IX, markers from TcChr30 (XM_806918 and XM_812638) hybridized with the band XII, and marker glucosamine-6-phosphate isomerase (TcChr35) mapped to the bands I and XI. Markers from these bands detected orthologous counterparts distributed over almost the entire length of chromosome Tb11 of *T. brucei*. In the same way, markers from chromosomal bands V and XVIII (e.g. XM_803658 of the TcChr7) and from part of the TcChr39 (katanin, chromosomal band XVI) were distributed throughout orthologous counterparts of *T. brucei* chromosome Tb9 of (Fig. 6C).

Analysis of TriTryp database comparative syntenic regions confirmed these data, validating synteny among Tb11 and several *T. cruzi* chromosomes (Fig. S4). The beginning of TcChr39 and the entirety of TcChr14, TcChr30 and TcChr35 are represented by arrows of yellow, brown, pink and purple, respectively. It is interesting to note that other chromosomal fragments are homologous to Tb11 (TcChr16, TcChr26, TcChr27 and TcChr41, black arrows). However, these TcChr were not analyzed in detail and the syntenic fragment size related to each chromosome is unknown.

## Discussion

### Differences in genome size in *T. cruzi* lineages

The absolute DNA content of *T. cruzi* epimastigotes was determined using total DNA isolated from a defined number of cells and a DNA-specific fluorescent dye. The presence of parasites in S or G2 phases of the cell cycle can lead to an overestimation of DNA content of non-multiplying cells when all measurements are averaged. To avoid this complication, the analysis was performed using parasites arrested in the G1 phase of the cell cycle by treatment with HU. Synchronization was confirmed by comparison between hydroxyurea-treated and non-treated parasites (Figure S1). During HU treatment, cells accumulate in the late G1 to early S phase of the cell cycle with a concomitant decrease in the G2 peak.

To estimate the nuclear genome size it was assumed that kDNA accounts for 20% of a parasite's total DNA and that the proportion of nuclear and kDNA was the same across strains [45], [53], [54]. The degree of accuracy of DNA measurements was confirmed by comparing the DNA content of haploid and diploid lineages of *S. cerevisiae*. The genome size estimated for *S. cerevisiae* (12.72 Mb) is almost identical to that obtained by whole genomic sequencing (12.07 Mb), confirming the accuracy of this technique. The DNA content estimated for the diploid lineage was 1.9-fold greater than the haploid lineages.

Overall, the mean nuclear genome size of the TcII, TcV and TcVI isolates was 125 Mb, and values for TcI isolates ranged from 89 Mb in Dm28c to 115 Mb in Tc1161 (Table 1). These values are comparable to those obtained by Lewis et al., 2009 who measured the genome size of 54 *T. cruzi* isolates using flow cytometry of fixed parasites stained with propidium iodide, and they are in agreement with previous findings that TcI strains have lower genome sizes than strains from other DTUs strains [17], [21], [26], [48], [55], [56]. The low variability in genome size values observed among the TcI isolates and among the TcII, TcV and TcVI isolates indicates that the dsDNA quantification technique was reliable and that the estimated genome sizes of the different isolates are accurate.

However, several points should be noted. Although the mean amount of DNA is homogeneous across the TcI group, some TcI strains (Tc1161 and 92101601P) had a DNA content 29% higher than the average, comparable to the TcII group [21], [57]. *T. cruzi* has been considered to be at least diploid, but aneuploid in hybrid strains [8], [17], [58].

Assuming a diploid content of 89.5 Mb for G and Dm28c, the mean DNA content of Tc1161 was 129% of the reference diploid value, very close to triploid cell (2.6n). One possible interpretation of this result would be that Tc1161 was generated by fusion of two TcI nuclei. The DNA content could be decreased with time, the mean genomic DNA content evolving from tetraploidy to near triploid. Its DNA content (115 Mb) could correspond to a loss of 45.5% of the DNA from the tetraploid nucleus (179 Mb). Recently, Minning et al. proposed “fusion then loss” mechanism occurs more frequently among *T. cruzi* strains than previously thought [9], [59].

It is interesting to note that 92101601P and Tc1161 strains have diverse origins; 92101601P was isolated from *Didelphis marsupialis* in the United States [21], [57] and Tc1161 (José-IMT) from a north-eastern Brazilian patient with end-stage chronic Chagasic cardiomyopathy [44]. The results presented herein agree with recent findings that sylvatic TcI populations are more genetically diverse than previously thought [21], [39], [41]. Recently, Cura et al. proposed a subdivision in TcI group (TcI a- TcI e) based on microsatellite motif of the intergenic spacer of Spliced-Leader gene relating to geographical distribution and transmission cycle [41] while Ocaña- Mayorga et al. identified geneticatlly distinct groups in TcI studying microsatellite data for ten variable loci [42]. Furthermore, in Tc1161 there are more chromosomal bands that are larger than in other TcI isolates, suggesting that the genome size changes could have occurred due to chromosomal aneuploidy. It is suggested that changes in genome size in TcI strains are relatively small and occur frequently, and involve gains of chromosomes. Karyotypic variations are frequent among trypanosomes and may be due to the non-disjunction of chromosomes at mitosis and the irregularity of genetic exchange in these organisms [38], [60].

The TcI group differs from other groups (TcII, TcV and TcVI) with regard to their genome size, and satellite DNA content is correlated with genome size for all isolates. This has not been accompanied by the simultaneous amplification of retrotransposons. The increase in genome size of TcII, TcV and TcVI isolates cannot be attributed solely to an increase in its satellite DNA amount. Other non-coding, repetitive DNA elements such as microsatellites, simple sequence repeats and large gene families of surface proteins can account for differences in genome size. In addition, karyotypic changes involving the gain of chromosomes were observed. Although the DNA content differs significantly among the *T. cruzi* lineages, the grouping of isolates by genome size agrees with the phylogenetic grouping, except for TC1161.

### Synteny, chromosome polymorphism and evolution

Regardless of chromosomal polymorphism, large syntenic groups are conserved among *T. cruzi* lineages (TcI, TcII, TcV and TcVI). Two large syntenic groups of 1.1 and 1.8 Mb in size (TcChr37 and TcChr39) were mapped to chromosomal bands XX and XVI of clone CL Brener, respectively. All specific markers demonstrated the same hybridization pattern in each isolate, suggesting the maintenance of gene order. Recently, strong synteny was confirmed by Sylvio X10/1 (TcI) genome sequencing data after comparison with CL Brener database [48]. Despite the large genetic distances that separate the lineages of *T. cruzi* [61], they exhibit conservation of chromosome structure. The syntenic regions are much larger than expected if rearrangements occur randomly, suggesting that they are conserved owing to positive selection. The results suggest a highly dynamic genome, which could be sorted into stable regions with genes coding for core activities, and dynamic regions where repetitive sequences and multigenic families are located. By array comparative genomic hybridization, Minning et al. described widespread Copy Number Variation among *T. cruzi* isolates [59]. It is more frequent in hot-spot sites where there are located a great number of repetitive elements and multigenic family in every chromosome [59].

The other syntenic group (TcChr7) was assigned to a single chromosomal band in the G strain, and two bands of similar size in isolates Esmeraldo-cl3, Dm28c and Tc1161 which could correspond to size-polymorphic homologous chromosomes. However, in other isolates (Y, CL Brener and SO3-cl5) the TcChr7 markers hybridized with two bands that differ greatly in size, 0.77 Mb (band V) and 2.5 Mb (band XVIII). The strong hybridization of the 0.77 Mb band with TcChr7 markers suggests that this band contains two homologous chromosomes of the same size. The weak hybridization signal of TcChr7 markers with the 2.5 Mb-band could be explained by the occurrence of a segment duplication in the 0.77 Mb-band followed by an insertion event in the larger 2.5 Mb-chromosome. By means of array comparative genomic hybridization, Minning et al. described that aneuploidies of chromosomes fragments are clearly evident [59]. The authors demonstrated a segmental aneuploidy in Brazil strain involving a 500 kb-fragment in the TcChr39 [59]. Herein, the duplicated chromosome-sized region was retained on both chromosomal bands as paralogous loci, increasing the dosage of several genes.

The results demonstrated that the integration of *in silico* assembled chromosome sequences and the molecular karyotype allowed the chromosomes present in the chromosomal bands to be identified and errors to be corrected, improving the quality of these complementary resources. This provides a valuable resource for comparative genomics of distinct *T. cruzi* lineages and between trypanosomatids.

Comparison of the genomes of *Trypanosoma* species is essential for identifying genetic changes involved in the acquisition of unique features in each species such as virulence factors (antigenic variation and antigenic variability), and developmental intracellular forms. By comparing the *in silico* assembled *T. cruzi* sequences with *T. brucei* chromosomes, homologous chromosomal regions in *T. brucei* would be defined. The chromosomeTb9 shares various regions of syntenic homology with *T. cruzi* chromosomes TcChr7 and 39, and chromosome Tb11 with TcChr14, 30, 35 and 39.

The mapping of the syntenic regions on *T. cruzi* chromosomal bands provides evidence for the occurrence of fusion and split events involving *T. brucei* and *T. cruzi* chromosomes. Specific markers for the *T. cruzi* chromosomes belong to syntenic regions on Tb9 and Tb11 chromosomes hybridized with distinct chromosomal bands in CL Brener. For instance, markers of Tb9 were mapped to *T. cruzi* chromosomal bands V, XVI and XVIII, whereas markers for Tb11 hybridized with bands I, VII, IX, XI, XII, and XVI. By comparing the sequences of large chromosomal fragments from *T. brucei, T. cruzi* and *L. major*, Ghedin et al. proposed two alternative hypotheses to explain the genomic architecture in trypanosomatids [52]. The first hypothesis assumes that the ancestor of trypanosomatids had large chromosomes similar to those observed in *T. brucei*. Therefore, two independent fragmentation events would have occurred, one in the lineage leading to *Leishmania* and another leading to *T. cruzi*. Alternatively, if the ancestral state corresponded to smaller chromosomes only one event had to occur, a chromosomal fusion in the lineage leading to *T. brucei*.

Available information cannot determine which of these two hypotheses is most likely. However, the results presented in this study favor the second hypothesis. The data confirmed the occurrence of fusion and split events involving *T. brucei* and *T. cruzi* chromosomes, and suggest that the common ancestor of trypanosomes had small chromosomes and a more fragmented genomic organization; during speciation these fragments joined in different combinations, forming different genomes at the same time. It is likely that there is a selective pressure to keep gene order, although several karyotypic changes could be genetically neutral.

In the present study, data from chromosomal mapping and karyotyping were integrated with genome sequence data. The integrated map facilitated draft genome assembly and is a valuable resource for comparative genomics of trypanosomatids.

## Supporting Information

Figure S1 **Flow cytometry analysis demonstrating DNA synchronization after HU treatment of** ***T. cruzi*** **epimastigotes.** Panels **A**, **B**, **C** and **D** present Flow cytometry analysis of propidium iodide-stained epimastigotes of G strain (TcI), Y strain (TcII), clone SO3-cl5 (TcV) and clone CL Brener (TcVI), respectively. Histograms of non-treated-parasites are presented on the left and those treated with 20 mM HU for 24 h are presented on the right. The number above the first peak corresponds to the percentage of cells in G1 phase and that above the second peak to the S/G2 phase.
(TIF)

Figure S2 **Mapping of repetitive element satellite DNA and L1Tc retrotransposons on chromosomal bands of various isolates.** Chromosomal bands of Y strain and clone Esmeraldo-cl3 (E) from TcII; SO3-cl5 (S) from TcV; clone CL Brener (C) from TcVI; and clone G strain, clone Dm28c (D) and Tc1161 (José-IMT) isolate (T) from lineage TcI were separated by PFGE, transferred to nylon membranes and hybridized with probes satellite DNA and L1Tc, shown at left and at right of the figure, respectively.
(TIF)

Figure S3 **Illustration of synteny between the chromosomal band XX of clone CL Brener and** ***T. brucei*** **chromosome 10 (Tb10).** **Panel A**) Schematic representation of a chromosome comprising the scaffolds TcChr37 and TcChr4 within chromosomal band XX. The complete sequences of chromosome-sized scaffolds TcChr37 and TcChr4 comprise 41.6% of the entire chromosome XX (3.27 Mb). **Panel B**) The beginning of TcChr37 (red rectangle, 1 Mb) aligned to a chromosomal segment located at the middle of Tb10 (positions 2 Mb to 2.8 Mb). The end of the TcChr37 (blue rectangle, 350 kb) shows similarity with the portion included between 1.43 Mb to 1.75 Mb of Tb10. The TcChr4 (green rectangle) comprises the middle of Tb10 where there is a large chromosome inversion. Alignment among homologous genomic regions of *T. cruzi* chromosomes TcChr37 and TcChr4, and *T. brucei* chromosome Tb10. Homologous genes are connected by grey lines. On the basis of their gene contents, telomeric regions are located in the beginning of TcChr37 and at the end of TcChr4. Telomeric sequences located at the extremities of chromosome XX are indicated by red and green triangles, respectively. Soft and dark grey blocks represent *T. cruzi* and *T. brucei* chromosomes, respectively.
(TIF)

Figure S4 **Overview of Tb11 compared with** ***T. cruzi*** **Esmeraldo genomic sequences using TriTrypDB comparative syntenic regions analysis.** Tb11 was assigned to different *T. cruzi* chromosomes. The beginning of TcChr39, TcChr14, TcChr30 and TcChr35 are represented by yellow, brown, pink and purple arrows, respectively. Fragments belonging to other TcChr are demonstrated with black arrows. As demonstrated in Fig. 6, the syntenic regions located in Tb11 are distributed in different *T. cruzi* chromosomes: TcChr39 was assigned to chromosomal band XVI, TcChr14 to bands VII and IX, TcChr30 to band XII and TcChr35 to bands I and XI. The gene identification and the accession number of each marker are indicated in Table S4.
(TIF)

Table S1 **Variance analysis (ANOVA) of genome sizes in** ***T. cruzi*** **isolates.**
(XLS)

Table S2 **Variance analysis (ANOVA) of Satellite DNA (A) and L1Tc retrotransposon (B) copy numbers in various** ***T. cruzi*** **isolates.**
(XLS)

Table S3 **Chromosome localization of Satellite DNA (A) and L1Tc retrotransposon (B) in various** ***T. cruzi*** **isolates.**
(XLS)

Table S4 **Chromosomal localization of** ***T. cruzi*** **markers used in hybridization experiments.**
(XLS)
