# Supplementary figures and images for: Genome Size, Karyotype Polymorphism and Chromosomal Evolution in Trypanosoma cruzi
Source: PLoS One. 2011 Aug 12;6(8):e23042. doi: 10.1371/journal.pone.0023042 (PMC3155523; doi:10.1371/journal.pone.0023042)

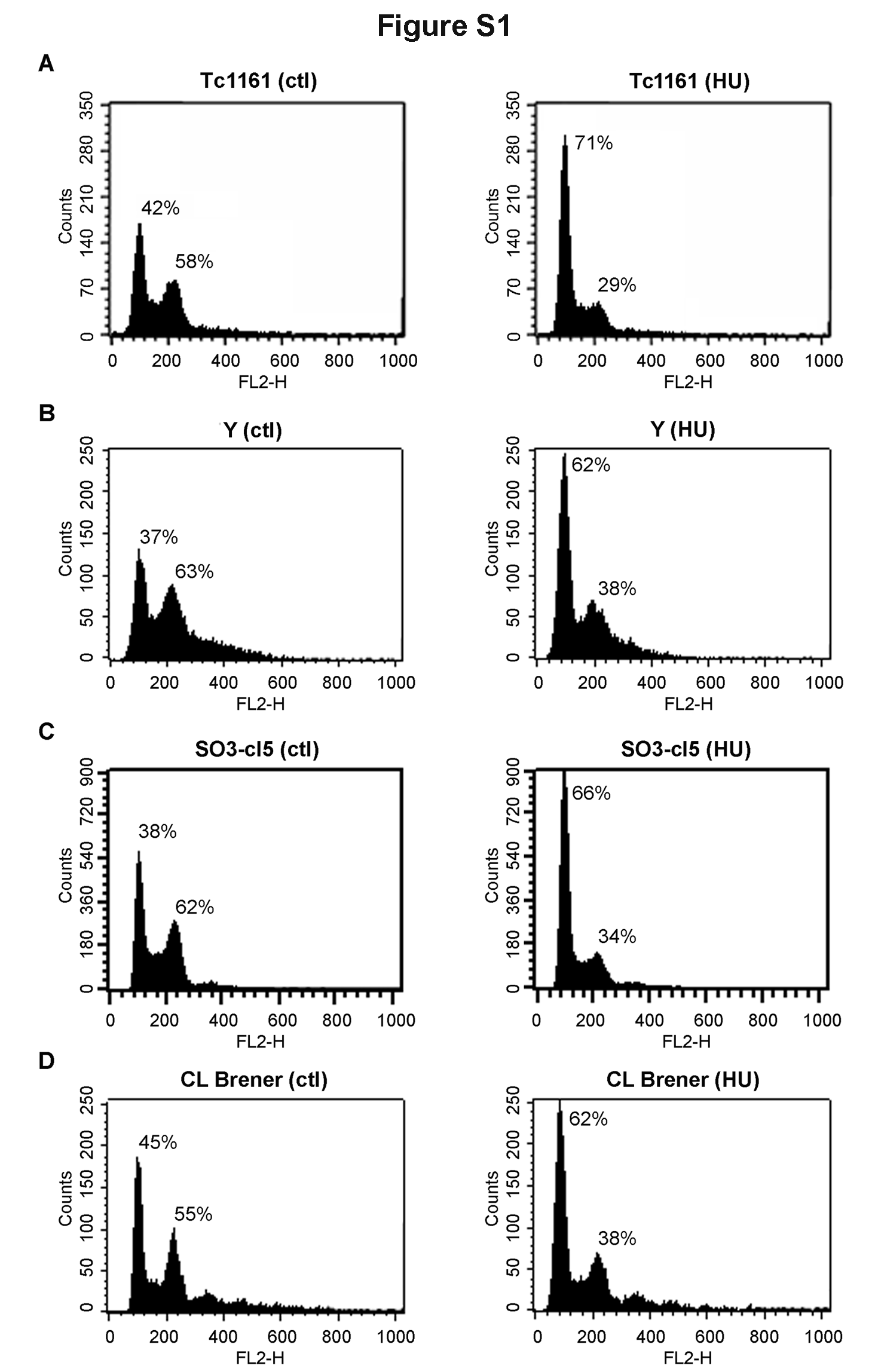

Supplement: Figure S1 — Flow cytometry analysis demonstrating DNA synchronization after HU treatment of T. cruzi epimastigotes. Panels A, B, C and D present Flow cytometry analysis of propidium iodide-stained epimastigotes of G strain (TcI), Y strain (TcII), clone SO3-cl5 (TcV) and clone CL Brener (TcVI), respectively. Histograms of non-treated-parasites are presented on the left and those treated with 20 mM HU for 24 h are presented on the right. The number above the first peak corresponds to the percentage of cells in G1 phase and that above the second peak to the S/G2 phase. (TIF) [file pone.0023042.s001.tif]

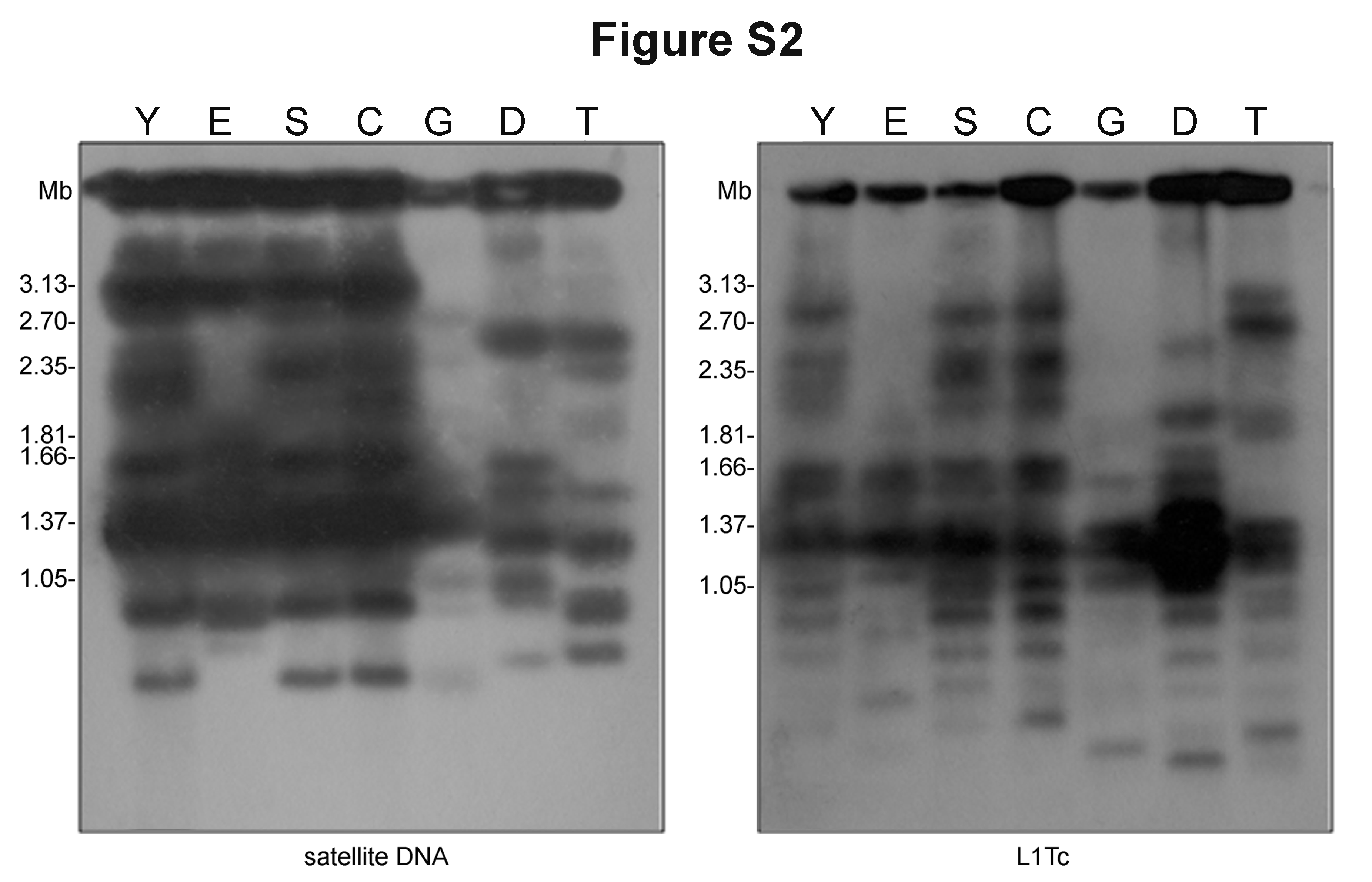

Supplement: Figure S2 — Mapping of repetitive element satellite DNA and L1Tc retrotransposons on chromosomal bands of various isolates. Chromosomal bands of Y strain and clone Esmeraldo-cl3 (E) from TcII; SO3-cl5 (S) from TcV; clone CL Brener (C) from TcVI; and clone G strain, clone Dm28c (D) and Tc1161 (José-IMT) isolate (T) from lineage TcI were separated by PFGE, transferred to nylon membranes and hybridized with probes satellite DNA and L1Tc, shown at left and at right of the figure, respectively. (TIF) [file pone.0023042.s002.tif]

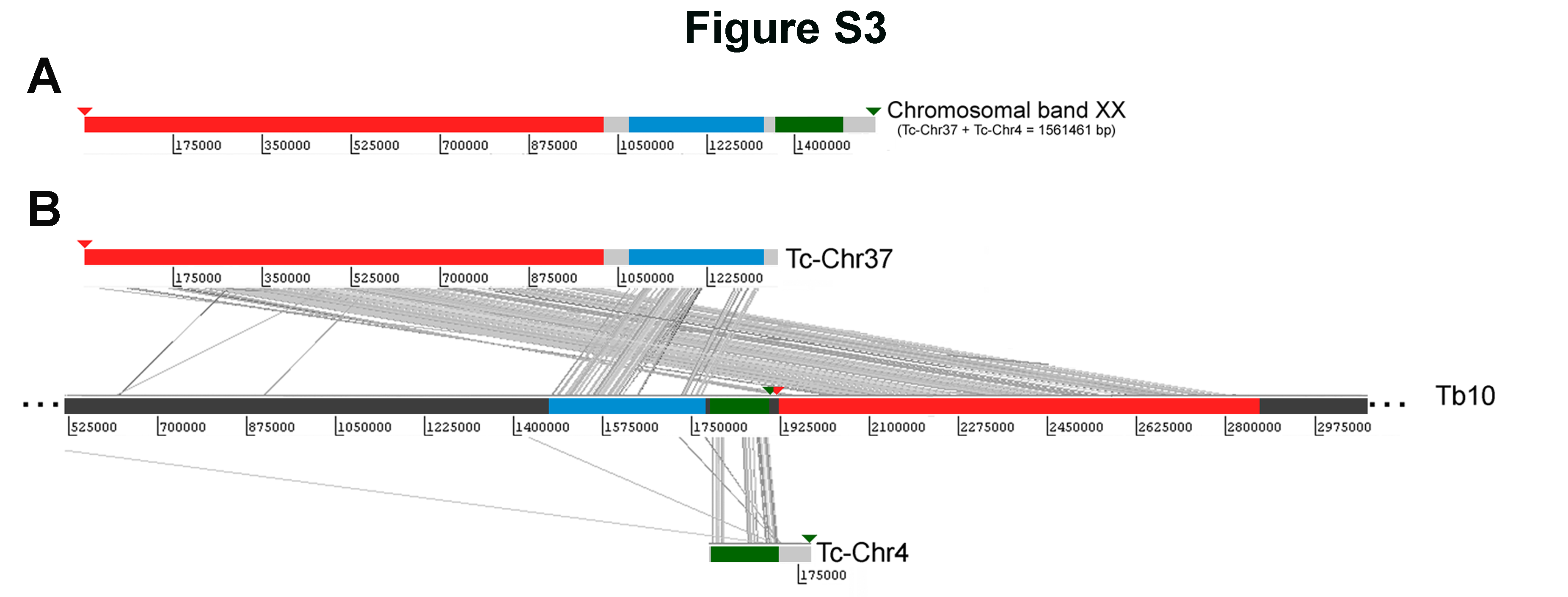

Supplement: Figure S3 — Illustration of synteny between the chromosomal band XX of clone CL Brener and T. brucei chromosome 10 (Tb10). Panel A) Schematic representation of a chromosome comprising the scaffolds TcChr37 and TcChr4 within chromosomal band XX. The complete sequences of chromosome-sized scaffolds TcChr37 and TcChr4 comprise 41.6% of the entire chromosome XX (3.27 Mb). Panel B) The beginning of TcChr37 (red rectangle, 1 Mb) aligned to a chromosomal segment located at the middle of Tb10 (positions 2 Mb to 2.8 Mb). The end of the TcChr37 (blue rectangle, 350 kb) shows similarity with the portion included between 1.43 Mb to 1.75 Mb of Tb10. The TcChr4 (green rectangle) comprises the middle of Tb10 where there is a large chromosome inversion. Alignment among homologous genomic regions of T. cruzi chromosomes TcChr37 and TcChr4, and T. brucei chromosome Tb10. Homologous genes are connected by grey lines. On the basis of their gene contents, telomeric regions are located in the beginning of TcChr37 and at the end of TcChr4. Telomeric sequences located at the extremities of chromosome XX are indicated by red and green triangles, respectively. Soft and dark grey blocks represent T. cruzi and T. brucei chromosomes, respectively. (TIF) [file pone.0023042.s003.tif]

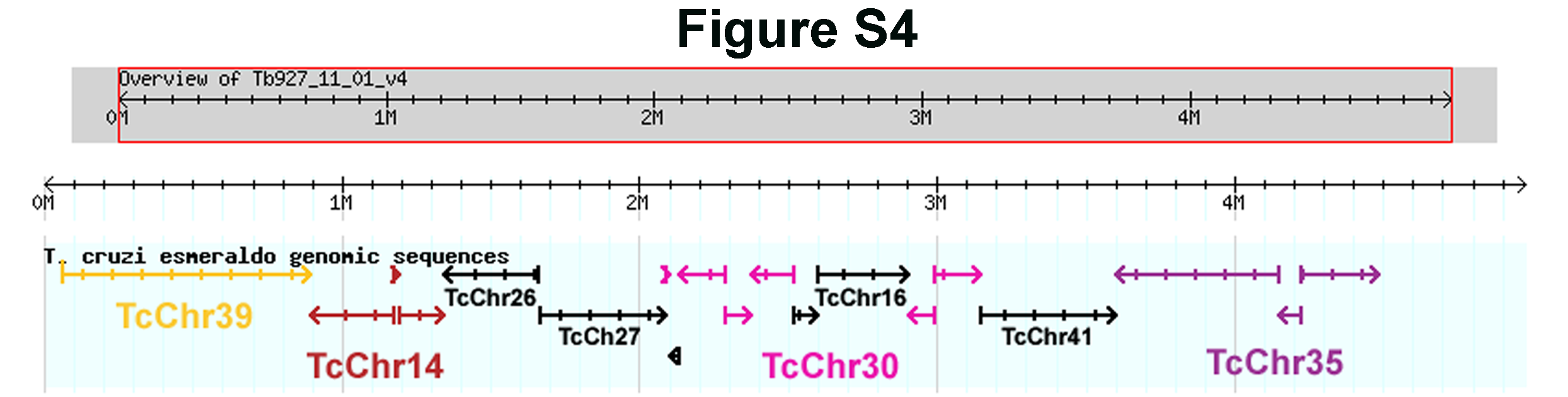

Supplement: Figure S4 — Overview of Tb11 compared with T. cruzi Esmeraldo genomic sequences using TriTrypDB comparative syntenic regions analysis. Tb11 was assigned to different T. cruzi chromosomes. The beginning of TcChr39, TcChr14, TcChr30 and TcChr35 are represented by yellow, brown, pink and purple arrows, respectively. Fragments belonging to other TcChr are demonstrated with black arrows. As demonstrated in Fig. 6, the syntenic regions located in Tb11 are distributed in different T. cruzi chromosomes: TcChr39 was assigned to chromosomal band XVI, TcChr14 to bands VII and IX, TcChr30 to band XII and TcChr35 to bands I and XI. The gene identification and the accession number of each marker are indicated in Table S4. (TIF) [file pone.0023042.s004.tif]
